# Unmasking Cyanide Toxicity: A Case of Encephalopathy and Ventilatory Support Following Smoke Inhalation

**DOI:** 10.7759/cureus.96859

**Published:** 2025-11-14

**Authors:** Bola Habeb, Darpan Kothia, Aaron Brooks

**Affiliations:** 1 Internal Medicine, Florida State University College of Medicine/Ascension Sacred Heart, Pensacola, USA; 2 Pulmonary and Critical Care Medicine, Ascension Sacred Heart, Pensacola, USA

**Keywords:** arterial blood gas (abg), cyanide poisoning, emergency toxicology, hydroxocobalamin, invasive mechanical ventilation, isopropanol, refractory lactic acidosis, smoke inhalation, toxic encephalopathy, venous blood gas (vbg)

## Abstract

Cyanide toxicity is an uncommon yet life-threatening consequence of smoke inhalation, particularly when synthetic materials burn in confined or poorly ventilated environments. Prompt recognition is essential, as delayed treatment can lead to rapid neurological decline and respiratory failure. We report the case of a 40-year-old incarcerated male who developed acute encephalopathy and respiratory compromise after exposure to dense smoke while working near a burning fuse in a prison yard. Worsening mental status and diminished respiratory effort necessitated endotracheal intubation and mechanical ventilation. Laboratory findings revealed a markedly elevated lactate level and a minimal arterial-venous partial pressure of oxygen (PaO₂) gradient, raising suspicion for cyanide poisoning. The patient demonstrated rapid clinical improvement following empiric administration of hydroxocobalamin, further supporting the diagnosis. This case underscores the importance of maintaining a high index of suspicion for cyanide toxicity in smoke inhalation victims, especially in high-risk, resource-limited environments such as correctional facilities. Early empiric administration of hydroxocobalamin may be life-saving and should not be delayed pending confirmatory testing.

## Introduction

Smoke inhalation remains a leading cause of morbidity and mortality in fire-related incidents, contributing to more deaths than direct thermal injury alone [1]. The toxic burden of inhaled smoke extends beyond hypoxemia and airway damage, as combustion of modern synthetic materials generates a wide array of poisonous gases. Among these, carbon monoxide (CO) and cyanide are the most clinically significant [2,3]. While CO poisoning is well-recognized and frequently screened for in emergency settings, cyanide toxicity is less often considered despite its potentially fatal consequences [4].

Cyanide is produced when nitrogen-containing materials such as plastics, polyurethane, vinyl, and wool are incompletely combusted [5]. Once inhaled, cyanide exerts its lethal effect by binding to cytochrome oxidase a₃ within the mitochondrial electron transport chain, halting oxidative phosphorylation and forcing anaerobic metabolism [6]. This cellular asphyxiation leads to impaired oxygen utilization, severe lactic acidosis, cardiovascular collapse, and rapid neurologic deterioration [7]. Inhalational exposure can result in death within minutes if left untreated [8].

Diagnosing cyanide poisoning in the acute setting is notoriously difficult. Laboratory confirmation (e.g., plasma or whole blood cyanide levels) is rarely available in real time, and clinical features often overlap with CO poisoning and other inhalational injuries [9]. Surrogate markers such as profound lactic acidosis, minimal arterial-venous oxygen gradient, and neurologic compromise in the context of smoke exposure can guide suspicion [3]. Because delays in treatment are associated with high mortality, empiric administration of antidotes such as hydroxocobalamin is recommended when cyanide toxicity is suspected [4,7]. Hydroxocobalamin binds cyanide to form cyanocobalamin (vitamin B12), which is renally excreted, offering a safe and effective intervention [8].

Despite its clinical relevance, cyanide poisoning from smoke inhalation remains underrecognized, particularly in environments where healthcare resources and diagnostic capabilities are limited. Correctional facilities, industrial sites, and poorly ventilated residential structures represent unique high-risk settings for such exposures. Here, we present the case of an incarcerated middle-aged male who developed encephalopathy and respiratory failure after smoke exposure in a confined outdoor setting. This case highlights the importance of maintaining a high index of suspicion for cyanide toxicity and illustrates the dramatic therapeutic impact of timely hydroxocobalamin administration.

## Case presentation

A 40-year-old incarcerated man with no known past medical history was found unresponsive outside his correctional facility workroom. He had been part of a work crew assigned to the facility’s agricultural section when he was discovered with a two-thirds-empty bottle of isopropyl alcohol nearby, prompting emergency medical services activation. On scene, the patient was noted to be unresponsive with decerebrate posturing and was subsequently intubated for airway protection before being transferred to an outside hospital. He received levetiracetam and benzodiazepines for concern of seizure activity, but showed no clinical improvement. The patient was then transferred to our institution’s intensive care unit (ICU) for a higher level of care.

Clinical findings

Upon arrival to the ICU, the patient was intubated, sedated, and mechanically ventilated, appearing acutely ill but hemodynamically stable. Vital signs were notable for a temperature of 37.6°C, blood pressure of 135/78 mmHg, heart rate of 112 beats per minute, respiratory rate of 14 breaths per minute, and oxygen saturation of 98% on assist-control ventilation (tidal volume 450 mL, positive end-expiratory pressure (PEEP) 5 cmH₂O, fraction of inspired oxygen (FiO2) 30%). Pupils were equal and reactive to light, and mucous membranes were moist without evidence of soot deposition around the mouth or nares. Skin examination revealed no cyanosis or cherry-red discoloration. Cardiovascular examination demonstrated tachycardia with a regular rhythm and no murmurs. Pulmonary auscultation revealed clear breath sounds bilaterally without wheezes, crackles, or rhonchi. The abdomen was soft, nondistended, and exhibited normoactive bowel sounds. Extremities were warm and well-perfused, without cyanosis or edema. Neurologic assessment was limited due to sedation and mechanical ventilation; however, brainstem reflexes were intact, pupils remained reactive, and no focal asymmetry was appreciated. When sedation was briefly minimized, intermittent non-purposeful movements and decerebrate posturing were observed.

Diagnostic assessment

The results of the initial laboratory workup are given in Table 1. 

**Table 1:** Laboratory data on admission pH: potential of hydrogen; pCO2: partial pressure of carbon dioxide; pO2: partial pressure of oxygen; BUN: blood urea nitrogen; AST: aspartate aminotransferase; ALT: alanine aminotransferase; INR: international normalized ratio; TSH: thyroid-stimulating hormone; HbA1C: glycated hemoglobin; FiO2: fraction of inspired oxygen * Abnormal lab values.

Parameters	Patient values on admission	Reference range, adults
pH	7.38	7.31–7.41
PCO2 (mmHg)	37	35–45
PO2 (mmHg)	178 on FiO2 30% *	60-80
Bicarbonate (mEq/dL)	22	22–29
Anion gap (mmol/L)	12	4-12
Carboxyhemoglobin %	8	0-3
Methemoglobin %	0.7	< 1.4
Oxyhemoglobin %	98	85-98
Lactic Acid (mmol/L)	5.3	0.5-2.2
Hemoglobin (g/dL)	14.9	12.0–15.5
Hematocrit (%)	41.9	34.9–44.5
White cell count (per mm3)	8000	3500–10500
Platelet count (per mm3)	255,000	150,000–450,000
Sodium (mEq/dL)	144	135–145
Potassium (mEq/dL)	4.5	3.5–5.1
BUN (mg/dL)	7	12–21
Creatinine (mg/dL)	0.74	0.72–1.25
ALT (units/L)	17	9–29
AST (units/L)	34	12–31
Alkaline Phosphatase (U/L)	86	40-150
Total bilirubin (mg/dL)	1	0.2-1.2
Ammonia (µmol/L )	39	15–45
INR	1	
TSH (mcIU/mL)	1.24	0.35–4.9
Blood glucose (mg/dL)	129	70-99
HbA1C	5.3	≤ 6.5
Ethanol level (mg/dL)	< 10	>299
Acetaminophen level (mcg/ mL)	< 3	< 30
Salicylate level (mg/dL)	< 5	5-29

**Figure 1:**
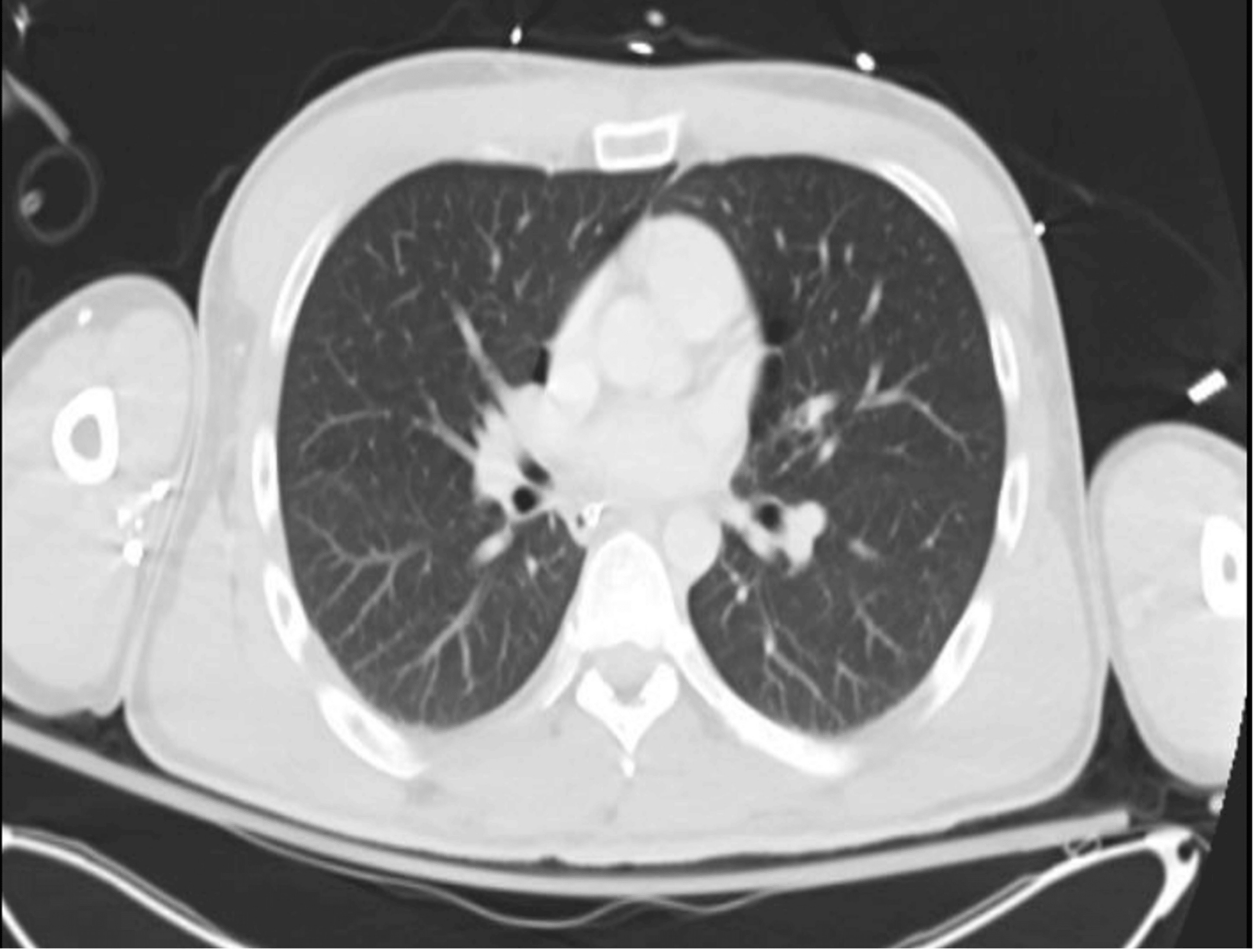
Non-contrast computed tomography of the chest demonstrating no acute abnormalities.

**Figure 2:**
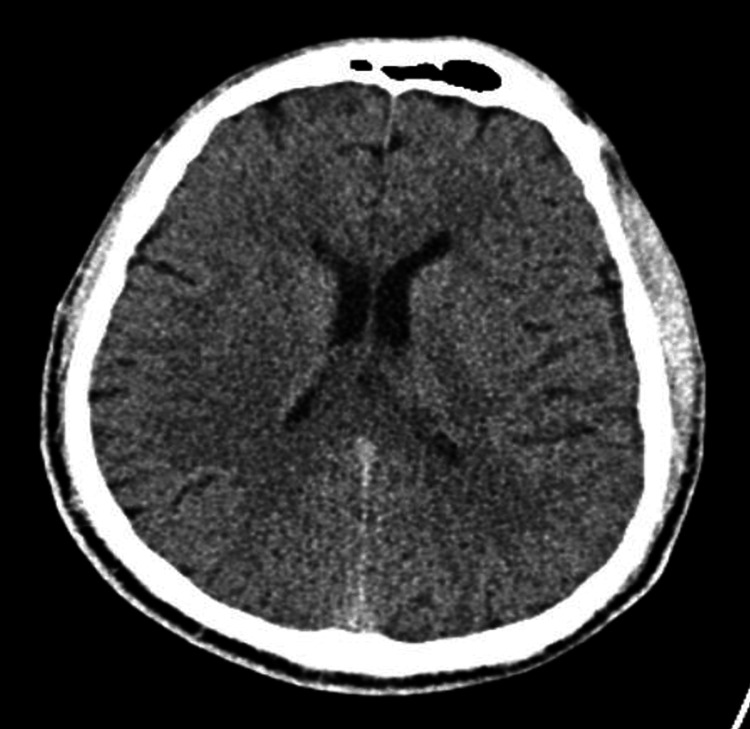
Non-contrast computed tomography of the brain showing no acute intracranial pathology.

**Figure 3:**
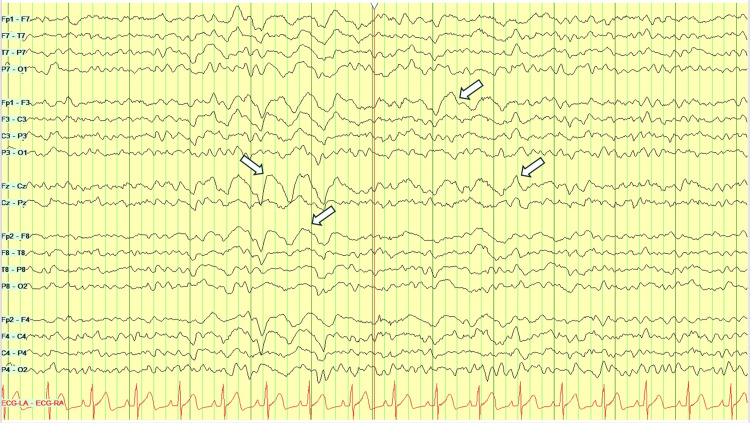
EEG showing intermittent generalized rhythmic delta activity (arrows), findings consistent with moderate encephalopathy and no epileptiform discharges. EEG: electroencephalogram

Urinalysis was unremarkable. The urine toxicology screen was positive for benzodiazepines, administered for concerns of seizures, and fentanyl, used for sedation. It was otherwise negative for methamphetamines, cocaine, opiates, phencyclidine, methadone, cannabinoids, and barbiturates. A non-contrast computed tomography (CT) scan of the chest was negative for a cardiopulmonary process (Figure 1). A non-contrast CT scan of the brain was unremarkable for acute intracranial pathology (Figure 2). A continuous video EEG revealed intermittent episodes of generalized rhythmic delta activity (GRDA), consistent with moderate encephalopathy, without any signs of epileptogenicity or electrographic seizure activity (Figure 3).

Critical thinking

Given the reported history of possible isopropyl alcohol ingestion, an osmolal gap was calculated and found to be within normal limits, as was the anion gap, making isopropyl alcohol intoxication a less likely cause of the patient’s presentation. Considering his occupational exposure in agricultural work, organophosphate poisoning was also entertained as a potential differential; however, the absence of characteristic findings such as excessive lacrimation, salivation, bronchorrhea, or diarrhea made this diagnosis less probable. Serum levels for methanol, isopropanol, ethylene glycol, and cholinesterase were subsequently ordered, though results were anticipated to take several days to return.

The patient’s serum lactate continued to rise despite adequate fluid resuscitation, increasing from 5.3 to 8.9 mmol/L. In view of the persistent metabolic derangement and lack of clinical improvement, simultaneous arterial and venous blood gas analyses were obtained, revealing a minimal arterial-venous oxygen gradient suggestive of impaired cellular oxygen utilization (Table 2).

**Table 2:** Comparison of simultaneous ABG and VBG parameters showing minimal oxygen gradient, suggestive of impaired cellular oxygen utilization. pCO2: partial pressure of carbon dioxide; pO2: partial pressure of oxygen; ABG: arterial blood gas; VBG: venous blood gas * Abnormal values.

Parameters	Arterial blood gas	Venous blood gas	Reference range, adults
pH	7.45	7.42	7.31–7.41
PCO2 (mmHg)	35	36	35–45 for ABG; 41-51 for VBG
PO2 (mmHg)	205 on FiO2 30% *	207 on FiO2 30% *	60-80 for ABG; 35-45 for VBG
Bicarbonate (mEq/dL)	22.3	22.8	22–29

With normal CO and methemoglobin levels demonstrated in Table 1, CO poisoning and methemoglobinemia were effectively excluded. Given the worsening lactic acidosis and the minimal arterial-venous oxygen gradient, cyanide poisoning was strongly suspected. Although a confirmatory cyanide level was sent, the result was expected to take several days; therefore, the patient was empirically treated with intravenous hydroxocobalamin 5 g. By the following day, the serum lactate had decreased to 0.6 mmol/L, and repeat simultaneous arterial and venous blood gases showed normalization of the oxygen gradient, consistent with restoration of effective cellular oxygen utilization (Table 3).

**Table 3:** Simultaneous ABg and VBG analysis following hydroxocobalamin administration demonstrated a normal arteriovenous oxygen gradient, consistent with effective cellular oxygen utilization. pCO2: partial pressure of carbon dioxide; pO2: partial pressure of oxygen; ABG: arterial blood gas; VBG: venous blood gas * Abnormal values.

Parameters	Arterial blood gas	Venous blood gas	Reference range, adults
pH	7.44	7.43	7.31–7.41
PCO2 (mmHg)	38	41	35–45 for ABG; 41-51 for VBG
PO2 (mmHg)	130 on FiO2 30% *	61on FiO2 30% *	60-80 for ABG; 35-45 for VBG
Bicarbonate (mEq/dL)	26	27	22–29

The patient’s level of consciousness and overall clinical status improved markedly, allowing for successful extubation. Further investigation revealed that he had been in close proximity to burning plastic materials before his presentation. He was transferred from the ICU to the medical floor on hospital day three and continued to demonstrate steady improvement, ultimately being discharged on day five. Prior to discharge, the patient was counseled on the hazards of smoke inhalation and advised on preventive measures to avoid future exposure.

A concise visual summary of the patient’s clinical timeline and progression is presented in Table 4.

**Table 4:** A concise visual summary of the patient’s clinical timeline and progression.

Phase	Event/Findings	Details
Exposure	Likely cyanide exposure from burning plastic materials	Patient was part of an agricultural work crew and found near burning plastic with an isopropyl alcohol bottle nearby.
Initial Deterioration (on scene)	Found unresponsive with decerebrate posturing	Emergency medical services noted no purposeful response; patient was intubated for airway protection.
Outside Facility Course	Treated empirically for seizure activity	Received levetiracetam and benzodiazepines; transferred to ICU for higher level of care.
ICU Admission	Sedated, mechanically ventilated; rising lactate	Initial lactate 5.3 mmol/L, increased to 8.9 mmol/L despite resuscitation; minimal arterial–venous O₂ gradient observed.
Diagnostic Evaluation	Toxicology and imaging workup performed	Normal CT brain and chest; EEG showed moderate encephalopathy; carboxyhemoglobin and methemoglobin normal.
Treatment	Empiric administration of hydroxocobalamin 5 g IV	Administered due to suspected cyanide poisoning; rapid improvement in lactate and oxygen utilization noted.
Recovery	Clinical and biochemical improvement	Lactate normalized (0.6 mmol/L); patient regained consciousness, successfully extubated, and transferred out of ICU on day 3.
Discharge	Full recovery	Discharged on day 5 with counseling on smoke inhalation prevention.

## Discussion

Cyanide toxicity represents one of the most insidious complications of smoke inhalation, with outcomes often hinging on the speed of recognition and initiation of therapy. Although CO poisoning is more frequently encountered and routinely screened for, cyanide may be equally or more lethal when unrecognized [1,2]. This case underscores the diagnostic and therapeutic challenges associated with cyanide intoxication in real-world, resource-limited environments such as correctional facilities.

Causes of cyanide poisoning

Causes of cyanide poisoning are diverse and extend beyond smoke inhalation, as summarized in Table 5.

**Table 5:** Causes of cyanide poisoning and reported clinical contexts.

Category	Examples	Reported Cases/Clinical Context	References
Environmental/Fire-related	Combustion of nitrogen-containing synthetic materials (plastics, polyurethane, vinyl, wool).	Cyanide is detected in smoke inhalation victims, often coexisting with CO poisoning in house or industrial fires.	[1,2,5,8]
Occupational	Electroplating, gold and silver extraction, metal polishing, and synthetic fiber production.	Acute toxicity and fatalities in workers due to inhalation of cyanide fumes or accidental ingestion during industrial processes.	[6,10,11]
Iatrogenic	Prolonged or high-dose sodium nitroprusside infusion; exposure to cyanide-containing compounds in labs.	Cyanide accumulation leading to lactic acidosis and altered mental status during prolonged nitroprusside therapy.	[12]
Intentional / Ingestion	Cyanide salts (e.g., potassium cyanide), cyanogenic glycosides from plants (cassava, apricot pits).	Case reports of suicide attempts and accidental ingestions; outcomes vary depending on the rapidity of antidotal therapy.	[4,9,10,11]

Pathophysiology and clinical features

The pathophysiology of cyanide toxicity centers on its inhibition of cytochrome oxidase a₃ within the mitochondrial electron transport chain, which halts oxidative phosphorylation and forces anaerobic metabolism [6]. The resulting mismatch between oxygen delivery and utilization leads to profound lactic acidosis, cardiovascular instability, and rapid neurologic decline [7]. Inhalational exposures are hazardous because cyanide is absorbed almost immediately via the pulmonary vasculature, producing life-threatening manifestations within minutes [8].

Diagnosis

Diagnosing cyanide poisoning remains clinically challenging, as confirmatory blood cyanide levels are rarely available in the acute setting [9]. Instead, surrogate clinical and biochemical markers often guide suspicion. Profound lactic acidosis (serum lactate >10 mmol/L), altered mental status, cardiovascular compromise, and the presence of minimal arterial-venous oxygen gradients in the context of smoke exposure are key diagnostic clues [2,4]. In this patient, severe lactic acidosis and a narrow PaO₂ difference between arterial and venous gases supported the diagnosis, consistent with prior literature [1,3]. Importantly, exclusion of other etiologies, such as isolated CO poisoning, requires careful clinical correlation, as both can coexist following smoke inhalation [5].

Management and therapeutic considerations

The management of cyanide poisoning focuses on rapid recognition, immediate supportive care, and prompt administration of specific antidotes. Initial measures include removal from the source of exposure, ensuring airway protection, providing 100% oxygen, and correcting metabolic acidosis [3]. Hemodynamic instability should be addressed with intravenous fluids and vasopressors as needed. The cornerstone of antidotal therapy is hydroxocobalamin (Cyanokit®; SERB Sàrl, Luxembourg), which binds cyanide to form cyanocobalamin (vitamin B₁₂) that is renally excreted [6,8]. It is preferred due to its rapid onset, favorable safety profile, and minimal interference with oxygen transport [3,7,8]. Alternative or adjunctive agents include sodium thiosulfate, which enhances the conversion of cyanide to thiocyanate via rhodanese, and sodium nitrite or amyl nitrite, which induce methemoglobinemia to bind cyanide, though the risk of worsening tissue hypoxia limits their use [4]. In severe or refractory cases, supportive modalities such as hyperbaric oxygen therapy or extracorporeal support may be considered. Early empiric administration of hydroxocobalamin remains the most effective strategy to prevent irreversible neurologic injury and death [6,8].

System-level considerations

From a systems perspective, this case highlights the unique vulnerabilities of under-resourced or high-risk environments such as prisons, industrial worksites, and residential structures with poor ventilation. Limited availability of diagnostics and antidotes can delay recognition and treatment, increasing the risk of fatal outcomes [2,5]. Early empiric therapy should be prioritized in such settings, given the minimal downside of hydroxocobalamin administration and the catastrophic risk of undertreatment [7]. Education of frontline providers, including those in correctional and emergency medicine settings, is crucial to improving recognition and timely management. Although the high cost of hydroxocobalamin (Cyanokit®) may deter routine stocking, this must be weighed against the potential loss of life and the substantially higher costs associated with prolonged hospitalization, intensive care utilization, and post-event investigations following delayed diagnosis. Strategic allocation of antidotes and protocols for rapid access in high-risk environments represents a cost-effective and life-saving systems intervention [13].

Clinical implications

Finally, this case reinforces the importance of maintaining a broad differential in smoke inhalation victims. While thermal injury, CO poisoning, and hypoxemia are common considerations, cyanide toxicity should remain high on the list when profound lactic acidosis, rapid neurologic decline, or hemodynamic instability are present [1,2,4]. Prompt empiric administration of hydroxocobalamin in such cases is not only justified but potentially life-saving.

## Conclusions

Cyanide toxicity remains an underrecognized but potentially fatal complication of smoke inhalation. This case illustrates the diagnostic challenges posed by overlapping features with other inhalational injuries, particularly CO poisoning, and highlights the value of surrogate markers such as severe lactic acidosis and minimal arterial-venous oxygen gradients. Importantly, it demonstrates the dramatic clinical response that can follow timely administration of hydroxocobalamin, even in resource-limited or high-risk settings such as correctional facilities.

Clinicians should maintain a high index of suspicion for cyanide poisoning in patients with altered mental status, respiratory compromise, and unexplained metabolic acidosis after smoke exposure. Early empiric treatment with hydroxocobalamin should be prioritized, as delays while awaiting confirmatory diagnostics can be fatal.
